# Extracellular cyclophilins A and C induce dysfunction of pancreatic microendothelial cells

**DOI:** 10.3389/fphys.2022.980232

**Published:** 2022-10-05

**Authors:** Rebeca Alvariño, Amparo Alfonso, Nadia Pérez-Fuentes, Jesús M. González-Jartín, Sandra Gegunde, Mercedes R. Vieytes, Luis M. Botana

**Affiliations:** ^1^ Departamento de Farmacología, Facultad de Veterinaria, Universidad de Santiago de Compostela, Lugo, Spain; ^2^ Grupo Investigación Biodiscovery, IDIS, Lugo, Spain; ^3^ Fundación Instituto de Investigación Sanitario Santiago de Compostela (FIDIS), Hospital Universitario Lucus Augusti, Lugo, Spain; ^4^ Departamento de Fisiología, Facultad de Veterinaria, Universidad de Santiago de Compostela, Lugo, Spain

**Keywords:** cyclophilin C, cyclophilin A, cyclophilin B, CD147 (EMMPRIN), endothelium, diabetes

## Abstract

Extracellular cyclophilins (eCyps) A and B are chemotactic mediators in several illnesses in which inflammation plays an important role such as diabetes and cardiovascular diseases. Recently, eCypC has been reported as a potential biomarker for coronary artery disease but its effect in endothelium has not been determined. Moreover, there is a lack of studies with all these proteins in the same model, which makes difficult a direct comparison of their effects. In this work, MS1 pancreatic microendothelial cells were treated with eCyps A, B and C and their impact on endothelial function was analysed. eCyps A and C stimulated the release of IL-6 and MCP-1 and increased the expression of the receptor CD147, but eCypB did not affect these pro-inflammatory markers. Moreover, eCypC activated the translocation of NFkB-p65 to the nucleus. All these effects were reversed by pre-treatment with cyclosporine A. eCyps also produced endothelial dysfunction, as evidenced by the decrease in eNOS activation. Finally, the crosstalk among eCyps addition and their protein and gene expression was evaluated. eCypA generated a depletion in its protein and gene levels, whilst eCyps B and C upregulated their own protein expression. Moreover, each eCyp altered the intracellular expression of other Cyps, including cyclophilin D. This work is the first report of eCyps influence on iCyps expression, as well as the first description of eCypC as an activator of CD147 receptor and a mediator of endothelial dysfunction, which points to a potential role of this protein in vascular complications associated to diabetes.

## Introduction

Diabetes-induced endothelial dysfunction is a critical factor in the initiation of micro- and macrovascular complications associated to this pathology. Along with the functional abnormalities found in large blood vessels, arterioles and capillaries are also affected by the damaging environment generated by chronic hyperglycaemia (Shi and Vanhoutte, 2017). Islets are comprised by a dense capillary network that plays a pivotal role in the pancreatic function, contributing to glucose sensing and insulin secretion. In fact, islet endothelial cells are supplied with 10% of total pancreatic blood flow, although they only account for 1–2% of pancreatic volume (Le et al., 2020). These cells provide oxygen, nutrients and a great diversity of signalling molecules with vasoactive, angiogenic, inflammatory and adhesion functions. Therefore, endothelial dysfunction has an important impact in the regulation of β-cells activity (Burganova et al., 2021). The most significant endothelium-derived mediator is NO, which has a key role in the maintenance of vascular homeostasis. NO contributes to pancreatic physiology by regulating secretion, vasodilation, and even cell proliferation and apoptosis (Hegyi and Rakonczay, 2011). Diminished NO production due to a reduction in endothelial NO synthase (eNOS) activity is the earliest and most crucial event of endothelial dysfunction (Maamoun et al., 2019).

Extracellular cyclophilin A (eCypA) is an inflammatory mediator that contributes to endothelial malfunction by affecting the expression of adhesion molecules such as E-selectin, activating mitogen-activated protein kinases (MAPK) and reducing eNOS activity (Jin et al., 2004; Kim et al., 2004; Xie et al., 2019). This protein is secreted by monocytes, lymphocytes, macrophages and endothelial cells themselves, and acts as a pro-inflammatory mediator, contributing to vascular complications (Xue et al., 2018). In fact, elevated CypA plasma levels have been detected in type 2 diabetes patients with coronary artery disease and have been correlated to diabetic nephropathy progression (Chiu et al., 2018; Ramachandran et al., 2014).

CypA belongs the cyclophilin (Cyp) family, composed by immunophilins with peptidyl-prolyl isomerase (PPIase) activity that catalyse the conversion of molecules from *cis* to *trans* conformation and show affinity for the immunosuppressant drug cyclosporine A (CsA) (Cao et al., 2019). Cyps are highly conserved proteins, with 18 isoforms located in different cellular compartments. This protein family includes cyclophilin B (CypB) and cyclophilin C (CypC), which are also released to the extracellular space. CypB is an endoplasmic reticulum (ER)-resident protein that participates in the regulation of the organelle homeostasis (Perrucci et al., 2015). It is secreted by different cell types such as lymphocytes or vascular smooth cells, acts as a chemotactic agent and its levels are increased in patients with hypertension, obesity and diabetes (Hoffmann and Schiene-Fischer, 2014; Zhang et al., 2017). CypC has been less studied, it is also mainly located in the ER and the Golgi apparatus and modulates ER homeostasis, but its specific physiological or pathological role remains elusive (Bukrinsky, 2015). Recent studies have described elevated serum levels of CypC as a promising biomarker for coronary artery disease (Alfonso et al., 2019; Bayón et al., 2021). Moreover, CypC release by human T lymphocytes and adipocytes has been reported, pointing to an important role of this isoform on vascular and metabolic diseases (Wang et al., 2004; Gegunde et al., 2021a).

Extracellular CypA (eCypA) and extracellular CypB (eCypB) activities are mediated by their binding to the receptor CD147, a ubiquitous expressed type I transmembrane glycoprotein of the immunoglobulin superfamily found in neutrophils, lymphocytes, endothelial and vascular smooth cells (Yurchenko et al., 2010). The interaction of eCypA with CD147 leads to the release of pro-inflammatory cytokines such as tumor necrosis-alpha (TNF-α) or interleukin-1 beta (IL-1β) *via* activation of nuclear factor kappa-light-chain-enhancer of activated B cells (NFkB). The effects of eCypB are contradictory, since its binding to CD147 induces extracellular signal-regulated kinase (ERK) activation and migration of T-cells and monocytes (von Ungern-Sternberg et al., 2018), but eCypB also reduced the release of pro-inflammatory mediators in macrophages activated with lipopolysaccharide (LPS) (Marcant et al., 2012). On the other hand, the receptor of extracellular CypC (eCypC) has not been identified, but it has been hypothesized that CD147 could be the signalling receptor of this isoform due to the great conservation of Cyps active site (Bukrinsky, 2015).

Although the role of extracellular and intracellular Cyps A and B in inflammation-related diseases has been described and both proteins share a common receptor, the potential effect of eCyps on their own intracellular levels has not been explored before. In a previous study, we observed a correlation among serum levels of eCyps and the intracellular expression of these proteins in T-lymphocytes from coronary artery disease patients (Gegunde et al., 2021b), which points to a possible crosstalk between high levels of eCyps and their expression in cells.

In this study, MS1 murine pancreatic microendothelial cells were treated with eCypA, eCypB and eCypC and their effects on endothelial dysfunction and CD147 expression were determined. Moreover, the crosstalk between elevated eCyps levels and their intracellular expression was evaluated. This is the first work that compares the effects of the three eCyps on the same cell line and describes the relationship among their extracellular and intracellular levels, as well as the first description of the potential role of eCypC on endothelial dysfunction.

## Material and methods

### Chemicals and solutions

Dulbecco’s Modified Eagle Medium (DMEM), fetal bovine serum, penicillin and streptomycin, 0.05% trypsin/EDTA, FITC-CD147 mouse monoclonal antibody, Pierce TM Protease Inhibitor Mini Tablets, phosphate buffered saline (PBS) (pH 7.2), Pierce TM Phosphatase Inhibitor Mini Tablets, Supersignal West Pico Luminiscent Substrate, Supersignal West Femto Maximum Sensitivity Substrate, oligo-dT primers, RevertAid Reverse Transcriptase and PowerUp SYBR Green Master Mix were purchased from Thermo Fisher Scientific (Madrid, Spain). Recombinant human CypA protein (endotoxin level <1.0 Eu/µg), CsA, anti-cyclophilin F, anti-sodium/potassium ATPase, anti-NFkB-p65 and anti-lamin B1 antibodies were from Abcam (Cambridge, UK). Recombinant human CypB (endotoxin level <1.0 Eu/µg) was from Antibodies-online and recombinant human CypC (endotoxin level <1.0 Eu/µg) was obtained from BioVendor (Brno, Czech Republic). Mouse High Sensitivity T Cell Magnetic Bead Panel, PVDF membranes, anti-phospho-eNOS (S1177), anti-eNOS and anti-β-actin antibodies were obtained from Merck (Madrid, Spain). Anti-CypA primary antibody and anti-CypB antibody were purchased from Elabscience (Madrid, Spain). Anti-CypC antibody was obtained from Proteintech (Manchester, UK). Sodium dodecyl sulphate polyacrylamide gels and Bradford reagent were purchased from Biorad (Hercules, CA, USA). Detachin™ solution was obtained from Genlantis (San Diego, US). Another reagent grade chemicals were obtained from Sigma-Aldrich (Madrid, Spain).

Lysis buffer used for protein extraction of membranous and cytosolic fractions was composed by 50 mM Tris HCl (pH 7.4), 150 mM NaCl, 1 mM EDTA and 1% Triton x-100, supplemented with a complete phosphatase/protease inhibitor cocktail. Hypotonic buffer used for nuclear and cytosolic lysates was composed by 20 mM Tris-HCl pH 7.4, 10 mM NaCl and 3 mM MgCl_2_, containing a phosphatase/protease inhibitor cocktail. Nuclear extraction solution was composed by 100 mM Tris-HCl (pH 7.4), 2 mM Na_3_VO_4_, 100 mM NaCl, 1% Triton X-100, 1 mM EDTA, 10% glycerol, 1 mM EGTA, 0.1% SDS, 1 mM NaF, 0.5% deoxycholate, and 20 mM Na_4_P_2_O_7_, containing 1 mM PMSF and a protease inhibitor cocktail.

### Cell culture

MS1 murine pancreatic endothelial cell line was obtained from American Type Culture Collection (ATCC), number CRL2279. Cells were cultured in DMEM with high glucose (4.5 g/L) supplemented with 5% fetal bovine serum, 100 U/mL penicillin and 100 μg/ml streptomycin. Cells were maintained at 37°C in a humidified atmosphere of 5% CO_2_ and 95% air and dissociated weekly using 0.05% trypsin/EDTA.

### Measurement of inflammatory mediators release

Due to the great range of eCyps levels reported in patients (among 7.8 pg/ml and 3 μg/ml) (Mutlu et al., 2017; Zhang et al., 2017; Alfonso et al., 2019), eCyps concentrations (0.25 and 0.5 μg/ml) were chosen based on previous works with endothelial cells and eCypA (Kim et al., 2004; Jin et al., 2004; Xue et al., 2017), taking into account that the selected doses had pathophysiological relevance. Moreover, Cyps are highly conserved proteins, human and mouse sequences have near-full homology, so effects of human Cyps on *in vitro* and *in vivo* rodent models provide valuable outcomes (Wang and Heitman, 2005; Kalinina et al., 2019; Cao et al., 2020). In this context, human recombinant proteins were used in order to mimic the pathological conditions observed in inflammation-based illnesses such as diabetes, cardiovascular and metabolic diseases.

MS1 cells were seeded in 96-well plates at 2 × 10^4^ cells per well and allowed to growth for 24 h. Then, cells were treated with eCypA, eCypB and eCypC at 0.25 and 0.5 μg/ml for 24 h. LPS at 1 μg/ml was used as positive control. To confirm that cell activation was produced by eCyps, cells were pre-treated with 0.2 µM CsA for 1 h followed by addition of immunophilins. After incubation, cell medium was collected and kept at -80°C until analysis. Experiments were carried out by duplicate three independent times (n = 3). Levels of interleukin-6 (IL-6) and monocyte chemoattractant protein-1 (MCP-1) were determined with a Mouse High Sensitivity T Cell Magnetic Bead Panel (Merck, Darmstadt, Germany), following manufacturer’s instructions. Fluorescence was analyzed with Luminex 200™ instrument and xPONENT^®^ software (LuminexCorp, Austin, TX).

### Determination of CD147 receptor surface expression

The expression of CD147 receptor was determined as previously described, with modifications (Gegunde et al., 2021a). MS1 cells were seeded in 12-well plates at 80% of confluence. After 24 h, endothelial cells were pre-treated with 0.2 µM CsA followed by addition of eCyps at 0.5 μg/ml for 24 h. LPS at 1 μg/ml was used as positive control. Then, MS1 cells were detached with Detachin™ solution, centrifuged at 2000 rpm and 4°C for 5 min, and washed twice with PBS/BSA. Next, cells were resuspended in PBS/BSA containing 5 μg/ml of FITC-CD147 mouse monoclonal antibody and incubated for 1 h at 4°C and 300 rpm. After incubation, cells were washed twice with PBS for 10 min at 4°C and 300 rpm, resuspended and analyzed. The fluorescence of 5,000 events was evaluated by flow cytometry using the ImageStreamMKII instrument (Amnis Corporation, Luminex Corp, Austin, US). Results were analyzed with IDEAS Application 6.0 software (Amnis Corporation, Luminex Corp, Austin, US). Three independent replicates were performed (*n* = 3).

### Protein extraction

MS1 cells were cultured in 12-well plates at 80% of confluence and allowed to settle down for 24 h. Cells were pre-treated with 0.2 µM CsA for 1 h and stimulated with eCypA, eCypB and eCypC at 0.5 μg/ml for 24 h.

Membranous and cytosolic protein fractions were obtained as previously described (Alvariño et al., 2021). Cells were washed with ice-cold PBS and 100 µL of lysis buffer were added to each well. Then, cells were sonicated, centrifuged at 3,000 rpm and 4°C for 15 min and the supernatant was collected as the cytosolic fraction. The pellet was homogenized in the same lysis buffer and samples were incubated on ice for 30 min, sonicated in intervals of 10 min, and finally centrifuged at 12000 rpm and 4°C for 30 min. The supernatant was separated as the membranous fraction. Protein concentration in both fractions was quantified with Bradford method.

For obtaining nuclear and cytosolic fractions, cells were washed with ice-cold PBS and 100 µL of hypotonic fraction were added. Cells were incubated for 15 min on ice and centrifuged at 3,000 rpm and 4°C for 15 min. The supernatant obtained was collected as the cytosolic fraction and the pellet was resuspended in the nuclear extraction solution. Samples were incubated on ice for 30 min with vortexing intervals of 10 min. Next, lysates were centrifuged at 14000 g and 4°C for 30 min and the supernatant was separated as the nuclear fraction. Cytosolic fraction was quantified with Direct Detect system (Merck Millipore) and nuclear protein concentration was determined by Bradford method.

All the assays were performed three independent times (*n* = 3).

### Western blotting

Electrophoresis was resolved in 4–20% sodium dodecyl sulphate polyacrylamide gels, containing 15 µg of cytosolic protein or 10 µg of membranous and nuclear fraction from each sample. Trans-Blot^®^ semi-dry transfer cell system was used for proteins transfer to PVDF membranes. The Snap i.d. system (Merck) was used for membrane blocking and antibody incubation. CD147 was detected with anti-CD147 antibody (1:5,000), phosphorylated eNOS was quantified with anti-phospho-eNOS (S1177) (1:1000) and its total levels were detected with anti-eNOS antibody (1:1000). NFkB-p65 was analysed with anti-NFκB-p65 (1:10000). CypA was detected with anti-CypA primary antibody (1:1000), CypB was recognized with anti-CypB antibody (1:1000), CypC was quantified with anti-CypC antibody (1:1000) and CypD was detected with anti-cyclophilin F primary antibody (1:1000). Protein band intensity was corrected using anti-β-actin (1:10000), anti-sodium/potassium ATPase (1:10000) and lamin B1 (1:1000) in cytosolic, membranous and nuclear fractions, respectively. The immunoreactive bands were detected with Supersignal West Pico Luminiscent Substrate and Supersignal West Femto Maximum Sensitivity Substrate. Diversity GeneSnap system and software (Syngene, Cambridge, U.K.) were used for protein bands detection. Experiments were performed three independent times by duplicate (*n* = 3). The specificity of anti-CypA, anti-CypB, anti-CypC and anti-CypD primary antibodies was checked before starting the experiments by loading 0.5 µg of human recombinant Cyps A, B, C and D in sodium dodecyl sulphate polyacrylamide gels and performing the protocol as described above.

### Quantitative PCR

MS1 cells were seeded in 12-well plates at 80% confluence and allowed to attach for 24 h. Then, cells were pre-treated with 0.2 μM CsA for 1 h and eCypA, eCypB and eCypC at 0.5 μg/ml were added for 24 h. Total RNA was obtained using the HighPurity™ Total RNA Purification Kit (Canvax Biotech, Córdoba, Spain), following the manufacturer’s instructions. RNA purity and concentration were determined with Nanodrop™ 2000 spectrophotometer (Thermo Fisher Scientific). cDNA was synthetized with 0.5 µg of RNA, oligo-dT primers and RevertAid Reverse Transcriptase, following manufacturer’s instructions. Quantitative PCR was performed using PowerUp SYBR Green Master Mix in a Step-One real-time PCR system (Applied Biosystems, Madrid, Spain). cDNA was amplified with specific primers for *CypA, CypB, CypC* and *CypD* (Table 1). Data were analysed with the Step-One software (Applied Biosystems). *Glyceraldehyde-3-phosphate dehydrogenase* (*GAPDH*) was used as normalization control (Alvariño et al., 2020). Relative quantification was carried out using ΔΔCt method using the control cells as calibrator. All experiments were carried out three independent times in triplicate (n = 3).

**TABLE 1:** Primer sequences used in qPCR. *CypA*: cyclophilin A; *CypB*: cyclophilin B; *CypC*: cyclophilin C; *CypD*: cyclophilin D.

Gene	Primer sequence
*CypA*	5′ GAGCTGTTTGCAGACAAAGTT 3′ 5′ CCCTGGCACATGAATCCTGG 3'´
*CypB*	5′ GGCTCCGTCGTCTTCCTTTT 3′ 5′ ACTCGTCCTACAGATTCATCTCC 3′
*CypC*	5′ TGACGGACAAGGTCTTCTTTGA 3′ 5′ CAGAGCCACGAAGTTTTCCAC 3′
*CypD*	5′ CTTCCACAGGGTGATCCCAG 3′ 5′ ACTGAGAGCCATTGGTGTTGG 3′

### Statistical analysis

Results are presented as mean ± SEM. Differences were evaluated by one-way ANOVA and Tukey’s post hoc test with Graph Pad Prism 8.0 software. Statistical significance was considered at *p < 0.05, **p < 0.01 and ***p < 0.001.

## Results

### eCyps A and C activate microendothelium and increase CD147 receptor expression

At first, the effects of eCyps on cell viability were determined. MS1 cells were treated with eCyps A, B and C at 0.25 and 0.5 μg/ml, LPS at 1 μg/ml and CsA at 0.2 μM for 24 h and cell viability was evaluated by MTT test. None of the treatments affected cell viability (Supplementary Figure S1), so the study was further continued.

**FIGURE 1:**
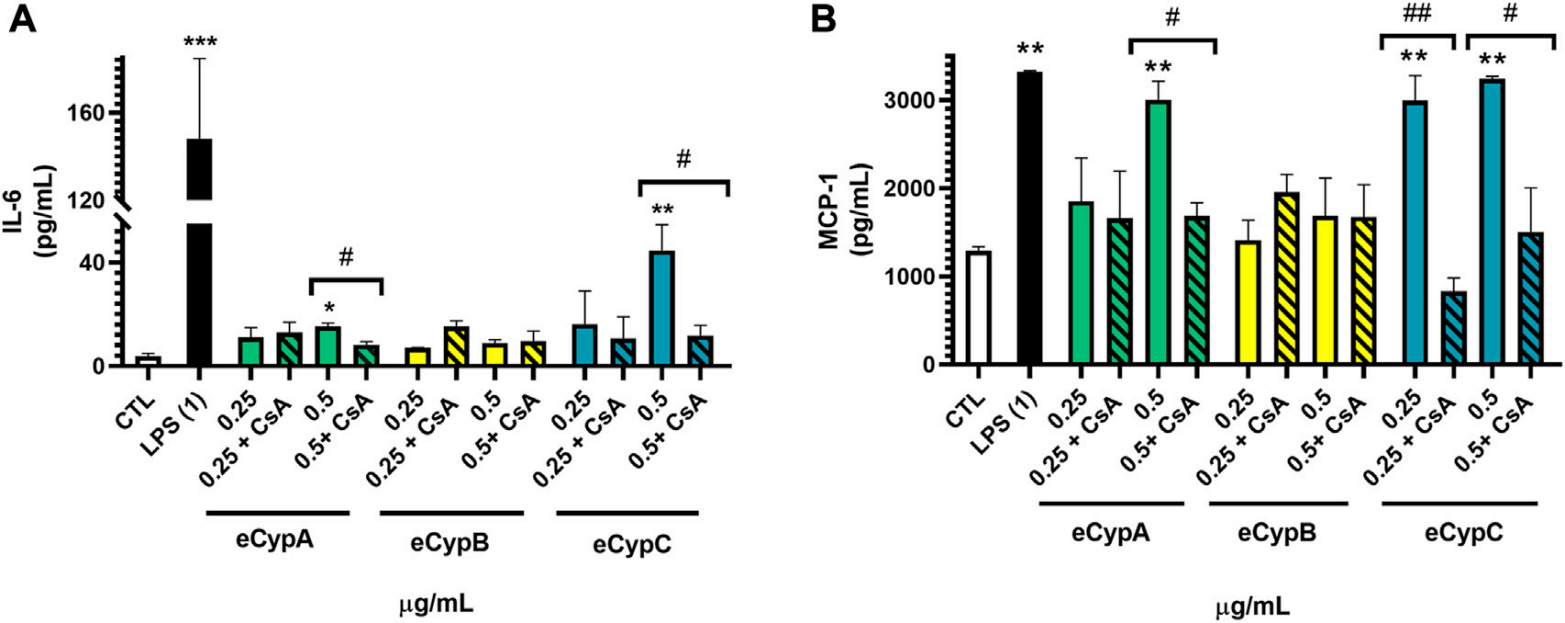
Effect of eCyps on inflammatory mediators release. **(A)** Levels of IL-6 in cell supernatant. **(B)** MCP-1 release of MS1 cells after eCyps addition. Endothelial cells were treated with eCyps A, B and C at 0.25 and 0.5 μg/ml for 24 h and levels of pro-inflammatory markers were assessed with a commercial multiplex kit. LPS at 1 μg/ml was used as pro-inflammatory control and pre-treatment with 0.2 μM CsA was performed to counteract eCyps effects. Results are mean ± SEM of three independent replicates. Statistical significance determined with one way ANOVA followed by post hoc Tukey’s test (*p < 0.05, **p < 0.01, ***p < 0.001, compared to control cells; #*p* < 0.05, ##*p* < 0.01 group comparison as indicated).

Next, MS1 pancreatic endothelial cells were treated with eCyps A, B and C as described above and the release of inflammatory mediators was monitored in cell supernatant. Levels of IL-6 and MCP-1 were evaluated with a commercial multiplex kit. Regarding IL-6, the greatest rise in its levels was produced by 1 μg/ml LPS (148.0 ± 36.7 pg/ml, *p* < 0.001). Addition of eCypA at the highest concentration also induced a significant increase in IL-6 release of 15.3 ± 1.3 pg/ml (*p* < 0.05). Treatment with eCypB did not exert any effect on the cytokine, but eCypC at 0.5 μg/ml produced an increment in IL-6 levels (44.6 ± 9.9 pg/ml, *p* < 0.01). When endothelial cells were pre-treated with 0.2 μM CsA for 1 h before stimulation with eCypA and eCypC, the augmentation in IL-6 levels was significantly reduced up to 8.3 ± 1.3 and 11.8 ± 3.9 pg/ml, respectively (*p* < 0.05) (Figure 1A). MCP-1 was also increased after treatment with the positive control LPS, reaching levels of 3,321.1 ± 15.5 pg/ml eCypA at 0.5 μg/ml and eCypC at both doses induced an augmentation in MCP-1 release among 2993.0 and 3,241.8 pg/ml (*p* < 0.01). Once again, eCypB did not produce an effect on the levels of the chemoattractant mediator. Pre-treatment with CsA significantly reversed the increase in MCP-1 generated by 0.5 μg/ml eCypA until 1684.8 ± 150.2 pg/ml, and reduced the levels of this chemokine after stimulation with eCypC at 0.25 and 0.5 μg/ml (829.7 ± 1542.4 and 1505.0 ± 501.5 pg/ml) (Figure 1B).

**FIGURE 2:**
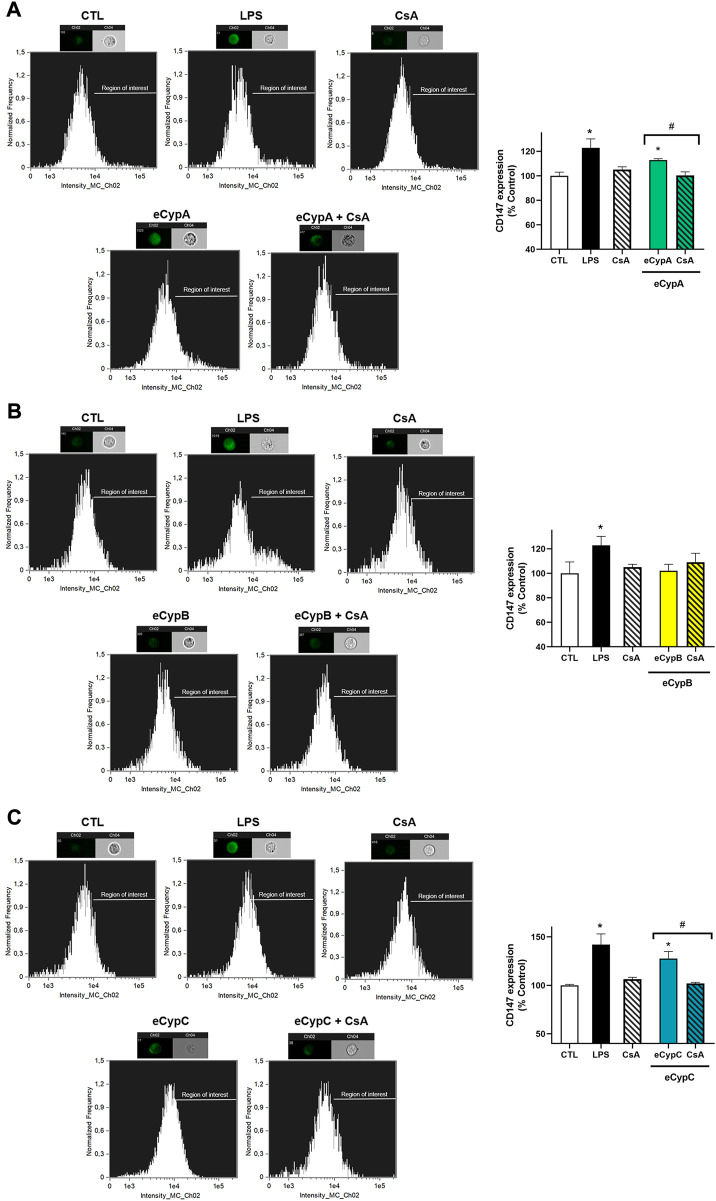
CD147 expression analysis by flow cytometry after eCyps treatment. Results of **(A)** eCypA, **(B)** eCypB, and **(C)** eCypC. MS1 pancreatic endothelial cells were treated with eCyps A, B and C at 0.5 μg/ml for 24 h and CD147 receptor expression in cell surface was determined with FITC-CD147 antibody. LPS at 1 μg/ml was used as pro-inflammatory control and pre-treatment with 0.2 μM CsA was carried out to revert eCyps effects. Upper panels show histograms of a representative replicate and lower panels present the corresponding graph bars. Data are mean ± SEM of three independent replicates, presented as percentage of control cells. Statistical differences were determined by one way ANOVA and Tukey’s tests (*p < 0.05, compared to control cells, #*p* < 0.05, pairwise comparison as indicated).

Next, the expression of CD147 receptor was analysed by flow cytometry. In view of previous results with eCyps, 0.5 μg/ml was the dose chosen for this assay. MS1 cells were pre-treated with 0.2 μM CsA for 1 h, followed by stimulation with eCypA, eCypB and eCypC for 24 h. Addition of LPS, the positive control, significantly increased CD147 expression of MS1 cells (Figure 2). With respect to eCypA, a slight augmentation of 112.9 ± 1.1% (*p* < 0.05) was observed when this immunophilin was added. This increase was reduced by pre-treatment with CsA (100.3 ± 2.8%, *p* < 0.05 compared to cells treated with eCypA) (Figure 2A). When eCypB was added to pancreatic endothelial cells, no effects were found on CD147 expression, agreeing with our previous results (Figure 2B). Finally, eCypC at 0.5 μg/ml significantly augmented the receptor expression, reaching a percentage of 117.1 ± 3.0% (*p* < 0.05). Pre-treatment with CsA decreased CD147 surface expression until 102.9 ± 3.8% (*p* < 0.05, compared to cells stimulated with eCypC) (Figure 2C).

**FIGURE 3:**
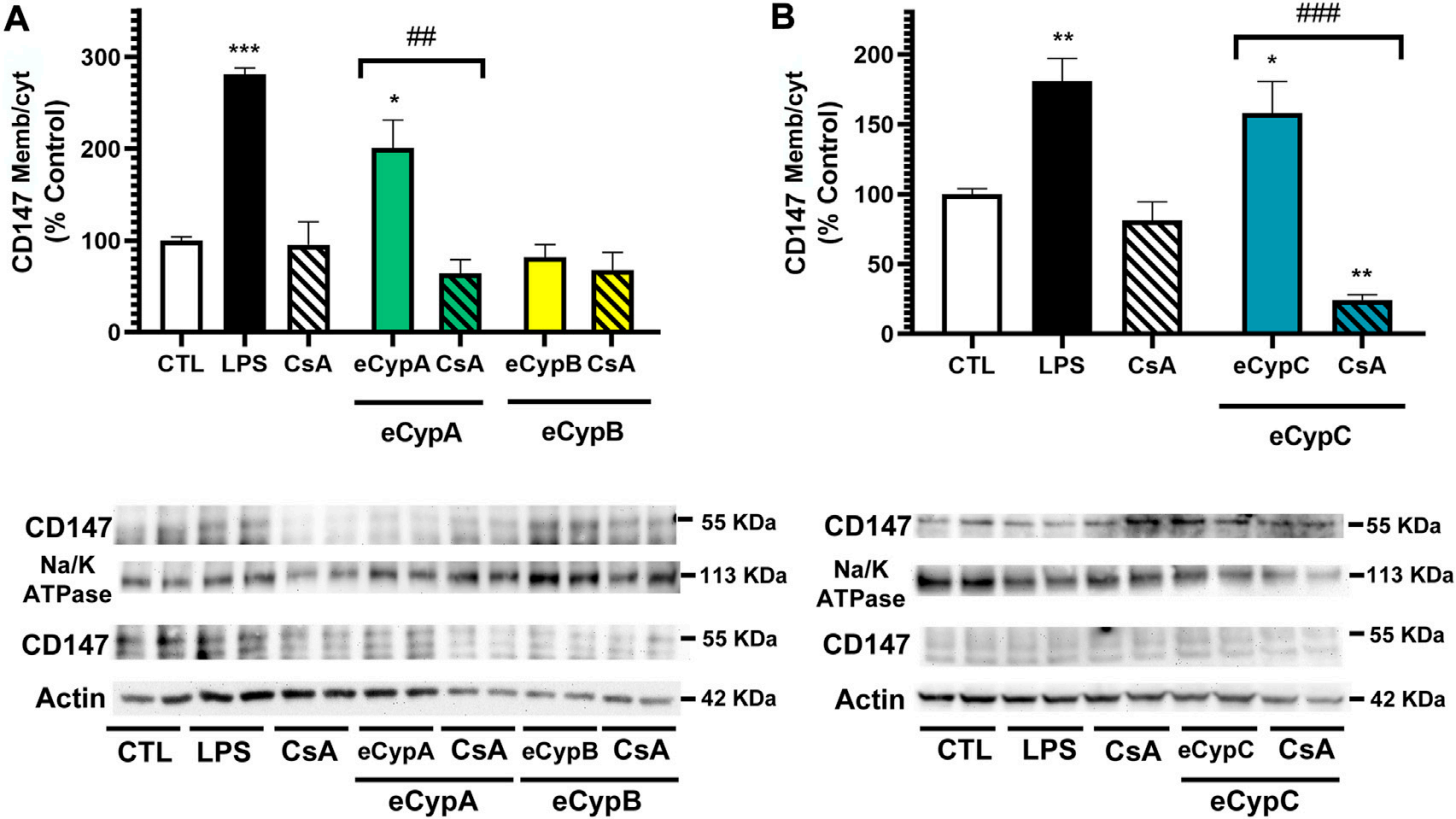
Analysis of CD147 expression by western blot. **(A)** Receptor expression after addition of eCypA and eCypB, **(B)** CD147 levels after eCypC treatment. MS1 cells were treated with eCyps at 0.5 μg/ml for 24 h and CD147 expression in cytosolic and membranous fractions was evaluated. CsA at 0.2 μM was added 1 h before treatment with 0.5 μg/ml eCyps. LPS at 1 μg/ml was used as positive control. Protein band expression was normalized by Na-K ATPase and actin levels in membranous and cytosolic fractions, respectively. The quantified bands are indicated by their molecular weight. Results are mean ± SEM of three replicates performed by duplicate, expressed as ratio between membranous and cytosolic expression. Statistical differences were determined by one way ANOVA and Tukey’s tests (*p < 0.05, **p < 0.01, ***p < 0.001 compared to control cells; ##*p* < 0.01, ###*p* < 0.001 pairwise comparison as indicated).

Since the differences detected by flow cytometry in CD147 expression, although significant, were small, the results were further confirmed by western blot. eCyps effects on the expression of glycosylated CD147, the active form, were evaluated. MS1 cells were treated as described above, lysed, and membranous and cytosolic fractions were separated for protein detection with a specific antibody. As Figure 3 shows, treatment with LPS induced a significant augmentation in CD147 expression in the membrane. eCypA addition also produced an increase in the translocation of the receptor to the membrane of 201.1 ± 30.3% (*p* < 0.05), which was counteracted when cells were treated with CsA (64.3 ± 14.9%, *p* < 0.01 compared to cells treated with eCypA). As expected, eCypB did not modify CD147 expression (Figure 3A). Regarding eCypC, this protein caused an intensification in the expression of the receptor in the membranous fraction, with levels of 169.4 ± 33.6% (*p* < 0.05). CsA reversed this effect, reducing CD147 levels up to 24.6 ± 5.4% (*p* < 0.001, compared to cells treated with eCypC) (Figure 3B).

### eCyps A, B and C affect eNOS and NFkB activation in pancreatic microendothelial cells

**FIGURE 4:**
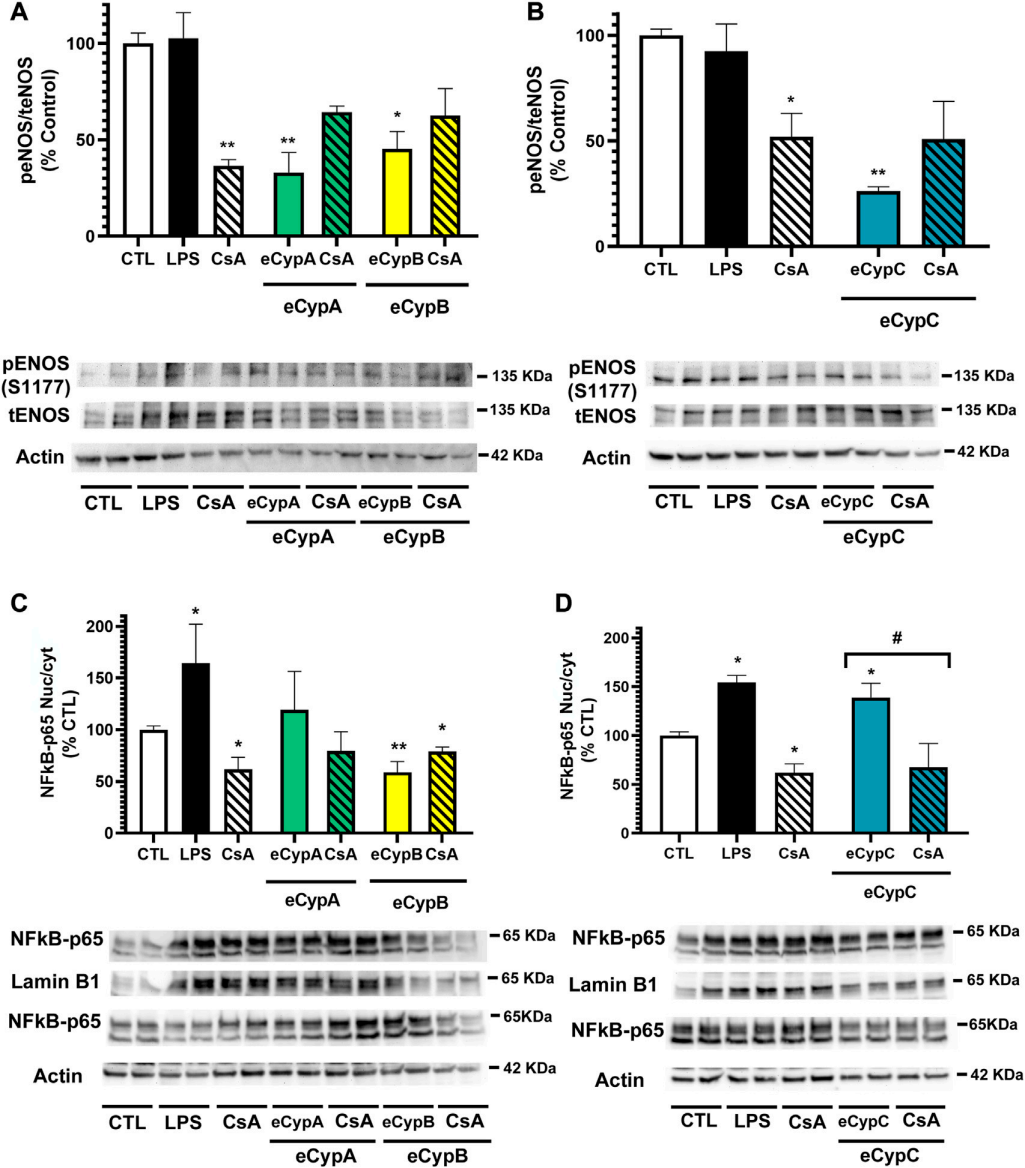
Analysis of eNOS and NFkB activation after eCyps A, B and C treatment. **(A)** Effect of eCypA and eCypB, **(B)** effect of eCypC on eNOS phosphorylation. **(C)** Expression levels of NFkB-p65 after eCypA, eCypB and **(D)** eCypC addition. Endothelial cells were pre-treated with 0.2 μM CsA for 1 h, followed by addition of eCyps at 0.5 μg/ml for 24 h eNOS activation was analyzed as the ratio between phosphorylated (S1177)/total protein levels. NFkB activation was measured as the ratio between nuclear and cytosolic levels. Protein band expression was normalized by lamin B1 and actin levels in nuclear and cytosolic fractions, respectively. The quantified bands are indicated by their molecular weight. Data are mean ± SEM of three independent experiments performed by duplicate and compared by one way ANOVA and Tukey’s tests (**p < 0.01 compared to control cells; #*p* < 0.05 pairwise comparison as indicated).

The study was followed by assessing if eCyps were affecting endothelial function, so the activation of eNOS, the enzyme responsible of NO release, was determined. With this purpose, phosphorylated and total eNOS expression was analysed (Liang et al., 2021). In this case, LPS did not affect eNOS activation, however, treatment with 0.2 μM CsA alone decreased the enzyme activation (Figures 4A,B). This effect of CsA on eNOS activity has been previously reported and is related to the effects of the drug on intracellular pathways (Arab et al., 2021; Harb et al., 2021). eCypA also reduced eNOS phosphorylation, showing levels of 33.0 ± 10.4% (*p* < 0.01). In this case, eCypB affected eNOS activation, reducing it until 45.3 ± 9.0% (*p* < 0.05). As expected, pre-treatment with CsA did not revert the inactivation produced by eCyps A and B due to the effects of the drug alone in the enzyme (Figure 4A). Addition of eCypC also produced a reduction in the phosphorylated status of eNOS (26.2 ± 2.1%, *p* < 0.01) (Figure 4B).

Next, the expression of NFkB was assessed. This complex is constituted by five subunits, namely p65, RelB, c-Rel, p50 and p52, which form homo and heterodimers that regulate the activity of the transcription factor. Particularly, the increase in p65/p50 dimers in the nucleus induces the expression of pro-inflammatory genes (Giridharan and Srinivasan, 2018). Therefore, the expression of NFkB-p65 was determined in nuclear and cytosolic fractions of MS1 cells after treatment with eCyps (Figures 4C,D). As expected, LPS produced a significant increase in p65 expression (*p <* 0.05), whilst CsA diminished NFkB activation (*p <* 0.05). eCypA augmented the transcription factor translocation to the nucleus (119.3 ± 30.1%), which was reduced up to 79.5 ± 18.5% by pre-treatment with CsA, but both effects did not reach statistical significance. On the other hand, eCypB decreased NFkB activation, showing levels of 58.9 ± 10.4% and 78.9 ± 4.3%, without and with CsA, respectively (Figure 4C). Regarding eCypC, an augmentation of 139.0 ± 14.6% (*p* < 0.05) in NFkB activation was observed, which was reverted by pre-treatment with CsA (*p* < 0.05) (Figure 4D).

### eCyps presence affects intracellular expression of Cyps A, B, C and D

**FIGURE 5:**
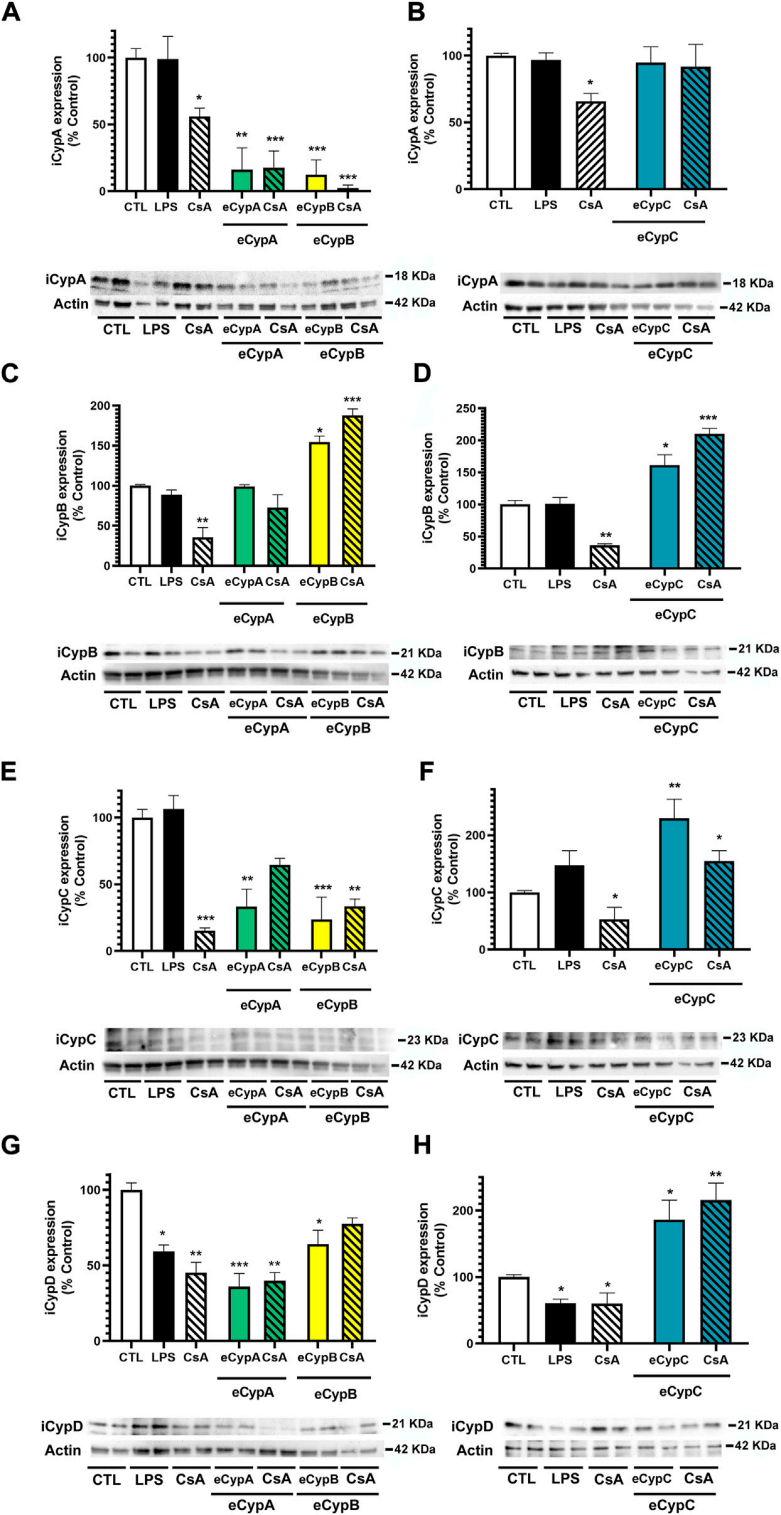
Determination of eCyps effects on intracellular expression of CypA, CypB, CypC and CypD. **(A)** iCypA levels after eCypA and eCypB addition. **(B)** Expression of iCypA when endothelial cells were treated with eCypC. **(C)** iCypB levels after treatment with eCypA and eCypB. **(D)** Effect of eCypC on intracellular expression of CypB. **(E)** iCypC expression levels after eCypA and eCypB addition. **(F)** eCypC effects on its intracellular levels. **(G)** Expression of iCypD in presence of eCypA and eCypB. **(H)** Levels of iCypD after treatment with eCypC. Protein band expression was normalized by actin levels. The quantified bands are indicated by their molecular weight. Data are mean ± SEM of three independent experiments performed by duplicate and expressed as percentage of control cells. Statistical significance evaluated by one way ANOVA and Tukey’s tests (*p < 0.05, **p < 0.01, ***p < 0.001, compared to control cells).

Next, the crosstalk between eCyps addition and the intracellular expression of Cyps A, B, C and D was evaluated. Due to the similarity among Cyps, the specificity of antibodies was tested before performing these experiments. With this objective, 0.5 μg of recombinant Cyps A, B, C and D were loaded in sodium dodecyl sulphate polyacrylamide gels and reactive bands were detected with the corresponding antibodies. Anti-CypA, anti-CypB and anti-CypC antibodies were specific to their corresponding targets, whilst anti-cyclophilin D (CypD) detected both CypD and CypA. Nevertheless, due to the different molecular weight of CypA and CypD (18 and 21 kDa, respectively), it was possible to distinguish both protein bands without uncertainty (Alvariño et al., 2022). Therefore, the expression of the four isoforms was analysed in MS1 cells. With respect to intracellular CypA (iCypA) levels, treatment with CsA generated a significant decrease, around 56.0 and 65.8% (*p* < 0.05) (Figures 5A,B). Surprisingly, a great decrease in iCypA levels was found after addition of eCypA and eCypB, which was not reverted by CsA (Figure 5A). In the case of eCypC, a different effect was found, since this isoform did not affect iCypA expression (Figure 5B). When intracellular CypB (iCypB) levels were analysed (Figures 5C,D), a reduction was observed with CsA treatment (35.5–36.3%), and a huge increase was produced by eCypB itself (154.4 ± 7.6%, *p* < 0.05) (Figure 5C) and eCypC (220.7 ± 11.5%, *p* < 0.05) (Figure 5D). Pre-treatment with CsA did not reduce this augmentation in any case.

Regarding intracellular CypC (iCypC) expression, the positive control CsA reduced the levels of this immunophilin in MS1 cells (Figures 5E,F). eCypA and eCypB addition also diminished iCypC levels to 33.2 ± 13.2% (*p* < 0.01) and 23.7 ± 16.7% (*p* < 0.001), respectively (Figure 5E). CsA slightly recovered the reduction produced by eCypA (64.4 ± 5.0%), but no effects were found in the case of eCypB. Figure 5F shows the results of eCypC addition in the intracellular levels of the protein itself, which were greatly increased until 229.8 ± 33% (*p* < 0.01). Pre-treatment with 0.2 μM CsA produced a decrease to 154.5 ± 18.5%, but it was not statistically significant. Finally, intracellular CypD (iCypD) expression was monitored in presence of eCyps A, B and C (Figures 5G,H). LPS and CsA significantly decreased the levels of the mitochondrial isoform among 59.4 and 45.1%. eCyps A and B also produced a decrease in CypD expression, with percentages of 36.1 and 64.0%, respectively, and CsA was not able to recover endothelial cells from this decrease (Figure 5G). With respect to eCypC, its addition resulted in an opposite effect, increasing iCypD levels both in the absence and presence of CsA (173.4 ± 24.4% and 215.7 ± 25.8%, respectively) (Figure 5H).

**FIGURE 6:**
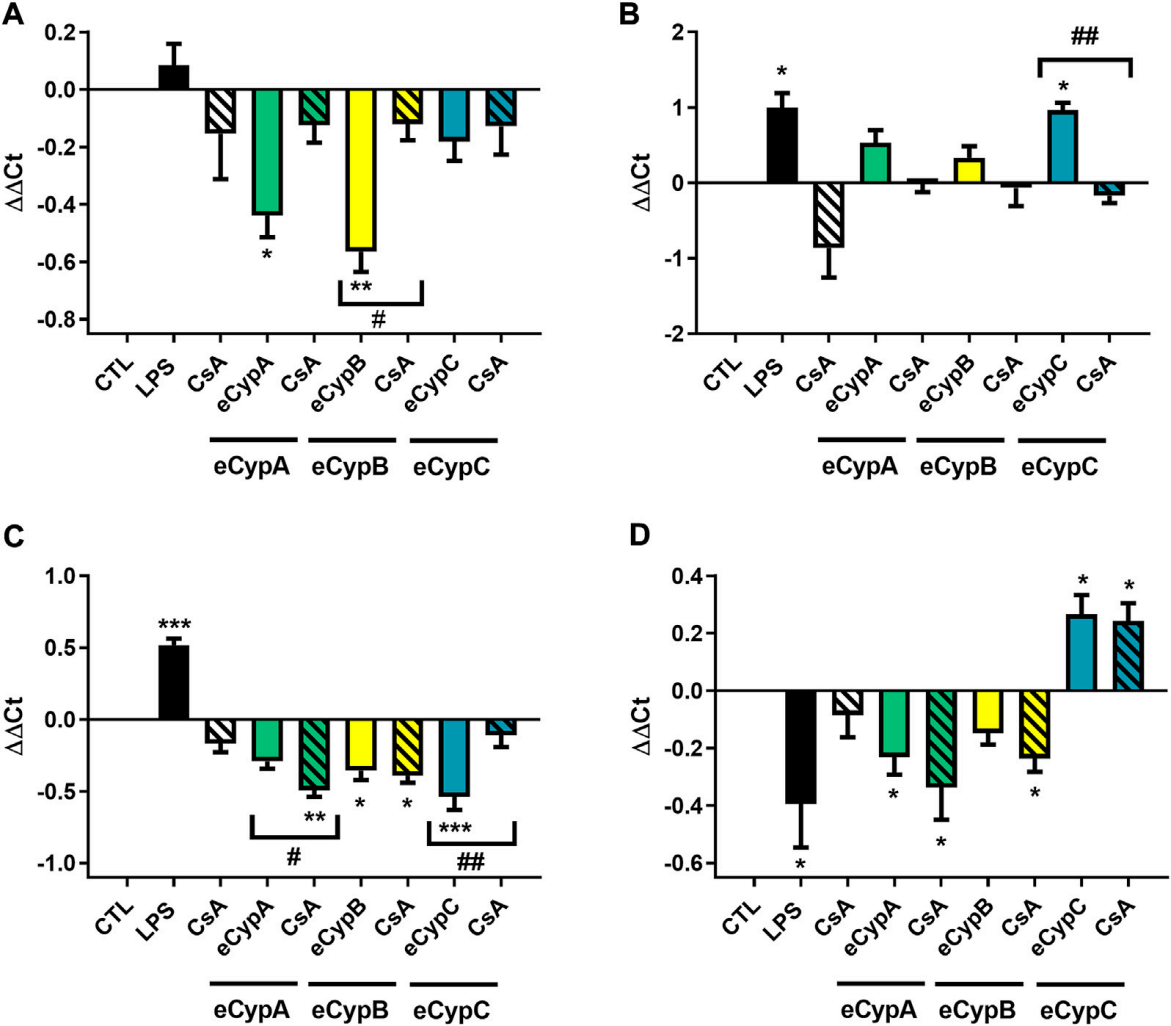
Gene expression of Cyps in presence of eCyps A, B and C. Relative gene expression of **(A)** *CypA*, **(B)** *CypB*, **(C)** *CypC*, and **(D)** *CypD* after addition of eCyps A, B and C at 0.5 μg/ml for 24 h. CsA at 0.2 μM was added 1 h before treatment and LPS (1 μg/ml) was used as positive control. Relative gene expression was calculated with ΔΔCt method. Control cells were used as calibrator and *GAPDH* as internal normalization control. Data are expressed as mean ± SEM of three independent replicates performed by triplicate. Statistical significance evaluated by one way ANOVA and Tukey’s tests (*p < 0.05, **p < 0.01, ***p < 0.001, compared to control cells; #*p* < 0.05, ##*p* < 0.01 pairwise comparison as indicated).

To further confirm the potential self-regulatory effect of eCyps, the relative expression of Cyps genes was determined (Figure 6). As Figure 6A shows, addition of eCyps A and B downregulated *CypA* expression, agreeing with western blot results. Pre-treatment with CsA reduced the effect of both eCyps, although statistical significance was only reached with the combination of eCypB and the immunosuppressant drug (*p* < 0.05). With respect to *CypB*, a significant increase was found in this gene expression when LPS and eCypC were added (*p* < 0.05), and CsA counteracted the effect of the immunophilin (*p* < 0.01) (Figure 6B). *CypC* expression was significantly decreased when eCypA and CsA were present in cell medium, as well as by treatment with eCypB and eCypC. The reduction induced by eCypC in its own expression was counteracted by pre-treatment with CsA (*p* < 0.01) (Figure 6C). Finally, *CypD* expression was downregulated by LPS, eCypsA and eCypB, and CsA did not revert this effect, as observed in western blot assays. In the case of eCypC, treatment with this protein upregulated the expression of the mitochondrial isoform (*p* < 0.05) (Figure 6D).

## Discussion

Cardiovascular diseases and diabetes mellitus are non-communicable illnesses that cause 19 million of deaths each year, being cardiovascular pathologies the first cause of mortality worldwide (Balakumar et al., 2016). Diabetic patients have among 2–4 times increased risk of presenting cardiovascular problems since they develop micro- and macrovascular complications associated to acute hyperglycaemia and oxidative stress augmentation (Godo and Shimokawa, 2017; Dal Canto et al., 2019). In this sense, eCyps have a pivotal role in both diseases, participating in atheroma plaque rupture and recruitment of inflammatory cells (Pahk et al., 2019), processes that are exacerbated in the presence of high glucose levels (Ramachandran and Kartha, 2012; Ramachandran et al., 2016).

This work describes for first time the ability of eCypC to induce endothelial dysfunction. The protein produced an increase in the secretion of pro-inflammatory mediators and in CD147 expression, reducing eNOS activity and activating NFkB translocation to the nucleus (Table 2). eCypC acted similarly to eCypA, which has been widely recognized as a key protein in several inflammation-related diseases because it acts as a chemokine and stimulates the release of pro-inflammatory factors (Xue et al., 2018). However, eCypC effect on IL-6 is lower than the produced by LPS addition, suggesting that the stimulation of endothelial cells by this protein is more related to a chemotactic activity. In fact, the increase in MCP-1 levels is similar to the generated by LPS. Regarding eCypA, it also increased the release of pro-inflammatory markers and NFkB-p65 expression was augmented in the nucleus, but statistical significance was not achieved. This could be related to the incubation time, as the transcription factor would be activated before the increase in cytokine secretion.

**TABLE 2:** Summary of eCyps effects in MS1 cells.

Assay/eCyp		eCypA	eCypB	eCypC
Release	IL-6	↑	—	↑
	MCP-1	↑	—	↑
Protein expression	CD147	↑	—	↑
	eNOS	↓	↓	↓
	NFkB	—	↓	↑
	iCypA	↓	↓	—
	iCypB	—	↑	↑
	iCypC	↓	↓	↑
	iCypD	↓	↓	↑
Gene expression	*CypA*	↓	↓	—
	*CypB*	—	—	↑
	*CypC*	—	↓	↓
	*CypD*	↓	—	↑

eCypA, extracellular cyclophilin A; eCypB, extracellular cyclophilin B; eCypC, extracellular cyclophilin C; IL-6, interleukin-6; MCP-1, monocyte chemoattractant protein-1; eNOS, endothelial nitric oxide synthase; NFkB, nuclear factor kappa-light-chain-enhancer of activated B cells; iCypA, intracellular cyclophilin A; iCypB, intracellular cyclophilin B; iCypC, intracellular cyclophilin C; iCypD, intracellular cyclophilin D. ↑, increase; ↓, decrease; -, no effect.

Based on the obtained results, it can be presumed that eCypC activity is mediated by the receptor CD147, since the analysis of its expression revealed an augmentation in the plasmatic membrane. Cyps present a highly conserved active pocket and direct interactions among Cyps A and B and CD147 have been demonstrated, so CypC is probably acting similarly. In fact, CypC and CD147 have been co-localized in breast epithelial cells (Mi et al., 2007). Although future experiments should be performed to confirm the direct interaction among eCypC and CD147 and to better understand the pathways affected by this protein, these results point to an important role of CypC in age-related pathologies in which chronic inflammation and endothelial dysfunction are implicated such as cardiovascular diseases or diabetes. In fact, CypC levels are increased in the serum of patients with coronary artery disease and have been correlated with risk factors such as hypertension, dyslipidemia and age (Alfonso et al., 2019; Bayon et al., 2020; Bayón et al., 2021).

Regarding eCypA, its effects on endothelial cells have been previously reported (Jin et al., 2004; Kim et al., 2004), but to our knowledge, microvascular cells had not been used so far. Our results confirm the damaging consequences of CypA presence in the extracellular milieu of pancreatic microendothelial cells. It has been previously described that eCypA affects islet cells, increasing the release of interleukins 5 and 17 (Li et al., 2006), and our data reinforce the importance of this protein in pancreas functioning and thus, in diabetes. CypA is released through a regulated vesicular pathway in vascular smooth muscle cells in which activation of Rho-associated protein kinase 2 (ROCK2) is involved (Satoh et al., 2010; Shimokawa and Godo, 2020). In endothelial cells, ROCK2 inhibits eNOS activity and increases MCP-1 expression augmenting monocyte migration (Takeda et al., 2019). In MS1 cells, high levels of eCypA resulted in a decrease of eNOS activation and a rise in MCP-1 release, so the potential involvement of ROCK2 in the effects observed should be assessed in future studies.

eCypB showed different effects on pancreatic microendothelial cells than those produced by eCyps A and C. The role of CypB in inflammation is controversial as opposite results have been reported. eCypB is a well-known chemotactic factor for leukocytes (Gegunde et al., 2021a), but its presence reduces LPS-induced inflammation and it is not able to stimulate cytokine secretion in macrophages (Marcant et al., 2012), agreeing with our results. Although eCypB signalling is mediated by CD147 receptor in many cell types, MS1 microvascular cells did not present an increased expression of the receptor. However, the immunophilin induced a decrease in eNOS activation that can be occurring through a CD147-independent pathway. Although the best described receptor of eCyps A and B is CD147, there are some effects of eCyps that cannot be only explained by their binding to CD147, so it is assumed that these proteins have other receptors (Xue et al., 2018). In fact, the triggering receptor expressed on myeloid cells-2 (TREM-2) has been recently identified as an eCypA target (Ji et al., 2021).

Finally, the crosstalk between eCyps presence and their intracellular levels was analysed. Surprisingly, when eCypA was added to microendothelial cells, its cytosolic protein expression was undetectable and pre-treatment with CsA did not counteract this effect. A similar effect was found when cells were treated with eCypB. These results were confirmed when gene expression was analysed, as *CypA* was downregulated after treatment with eCyps A and B, suggesting that both Cyps could have common signalling pathways. It has been described that reactive oxygen species (ROS) increase eCypA release in a ROCK2-dependent manner and high levels of eCypA stimulate ROS production, thus generating a vicious circle of increased oxidative stress (Satoh et al., 2010). In this context, as mentioned before, it would be interesting to analyse ROCK2 activation after eCyps addition, since eCypA could be also increasing its own release and enhancing the damaging environment surrounding endothelial cells. On the other hand, eCyps A and B could be affecting a transcriptional regulator of iCypA expression. The transcription factor signal transducer and activator of transcription 3 (STAT3), the proteasome modulator REGγ and Forkhead box protein O1 (FoxO1) have been reported as inducers of iCypA expression, so the effects of eCyps A and B on these pathways should be explored (Xie et al., 2020). Due to the addition of recombinant proteins to cell supernatant, it was not feasible to measure Cyps release to cellular medium, so it is possible that eCyps A and B treatments may be increasing the release of iCypA, making its intracellular levels undetectable.

With respect to the other Cyps expression, eCypA did not affect iCypB but it decreased iCypC and iCypD protein levels, as well as their gene expression. eCypB also downregulated the expression of iCypC and iCypD in a lesser extent than iCypA, whereas it increased its own expression. The same pattern was observed in qPCR assays, but only the effects on *CypC* were significant. Regarding eCypC, it seems to act in a different way, as iCypA levels were unaltered after addition of this isoform, but iCypB, iCypC and iCypD expression was upregulated. In this case, when gene expression was analysed, the results agreed with western blot experiments except for *CypC*, which was downregulated. In summary, eCyps A, B and C affect their own intracellular expression, so it appears that there are feedback regulatory processes involved in their pathological role. In addition, these proteins alter the protein and gene expression of other members of the Cyp family, pointing to common signalling pathways implicated is this modulation. eCyps A and B binding to CD147 triggers shared routes such as ERK (Kim et al., 2012), so it is presumably that eCypC could be also affecting this enzyme.

CypD is the only mitochondrial isoform of this chaperone family and modulates mitochondrial permeability transition pore (mPTP) opening. The pore is a regulator channel of mitochondrial homeostasis that under pathological conditions is massively opened and leads to cell death (Bernardi et al., 2021). mPTP modulation and thus, CypD, have been shown to play a role in endothelial resistance to oxidative stress, mitochondrial function and angiogenesis (Marcu et al., 2015; Peng et al., 2015). In MS1 cells, eCyps A and B addition produced a decrease in iCypD levels, so the presence of these proteins in cell medium seems to be conferring cell death resistance to microendothelial cells. Moreover, ERK is a known modulator of CypD, its activation reduces mPTP opening, so the previously mentioned effect of eCyps A and B on ERK might be related to the decrease in CypD expression (Porter et al., 2018). On the other hand, eCypC displayed an opposite effect on iCypD levels, it generated an augmentation in its expression. This effect on CypD points to a pro-apoptotic effect of eCypC, although it did not reduce endothelial cells viability.

The crosstalk observed among eCyps addition and the alteration of their own intracellular expression, as well as other isoform levels, points to common pathways involved in the modulation of this protein family that should be further studied to better clarify the complex role of these chaperones in cell physiology.

In conclusion, this work is the first study that allows a direct comparison between the three eCyps in the same cell line, as well as the first description of eCyps B and C effects on endothelial cells. Results indicate that eCypC acts similarly than eCypA, inducing pro-inflammatory mediators release, activating CD147 and generating endothelial dysfunction. eCypB presented different outcomes, without affecting neither the receptor nor cytokines release. Finally, it is evidenced a relationship among eCyps and iCyps levels, providing new insights into the intricate function of these proteins. These results in pancreatic microendothelial cells strengthen the crucial role of Cyps in the vascular dysfunction observed in diabetes, pointing to CypC as a potential mediator in this pathology.

## Data Availability

The raw data supporting the conclusions of this article will be made available by the authors, without undue reservation.
