# Supplementary material for: Extracellular cyclophilins A and C induce dysfunction of pancreatic microendothelial cells
Source: Front Physiol. 2022 Oct 5;13:980232. doi: 10.3389/fphys.2022.980232 (PMC9579281; doi:10.3389/fphys.2022.980232)

Supplementary Material

1. Materials and Methods

1.1 Cell viability

MS1 cells were seeded in 96-well plates at 2 x 10^4 cells per well and allowed to growth for 24 h. After this time, cells were treated with eCypA, eCypB and eCypC (0.25 and 0.5 µg/mL), LPS (1 µg/mL) and CsA (0.2 µM) for 24 h. Cell viability was determined with MTT (3-(4, 5-dimethyl thiazol-2-yl)-2, 5-diphenyl tetrazolium bromide) assay. After incubation, the plate was washed three times with Locke's buffer (154 mM NaCl, 5.6 mM KCl, 1.3 mM $CaCl_2$, 1 mM $MgCl_2$, 5.6 mM glucose and 10 mM HEPES, pH 7.4) and 500 µg/mL MTT was added to each well. Cells were incubated for 1 h at 37º and 300 rpm in an orbital shaker. Finally, MS1 cells were disaggregated with 5% sodium dodecyl sulphate and absorbance was read at 590 nm in a Synergy 4 microplate reader (BioTek instruments, Vermont, USA). Saponin from quillaja bark at 40 mg/mL was used as death control and its absorbance was subtracted from the other data. The experiments were carried out three times in triplicate.

2. Results

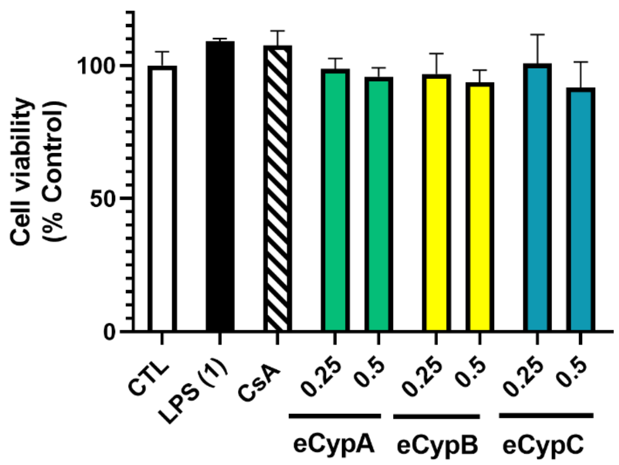

Supplementary Figure 1. Effect of eCyps on cell viability. Cells were treated with eCypA, eCypB, eCypC (0.25 and 0.5 µg/mL), LPS (1 µg/mL) and CsA (0.2 µM) for 24 h and their effect on cell viability was analysed with MTT test. Mean ±SEM of three experiments performed by triplicate. Data expressed as percentage of control cells and compared by one-way ANOVA and Dunnett's tests

3. Original Blots

-Figure 3A

- **CD147 (membrane)**

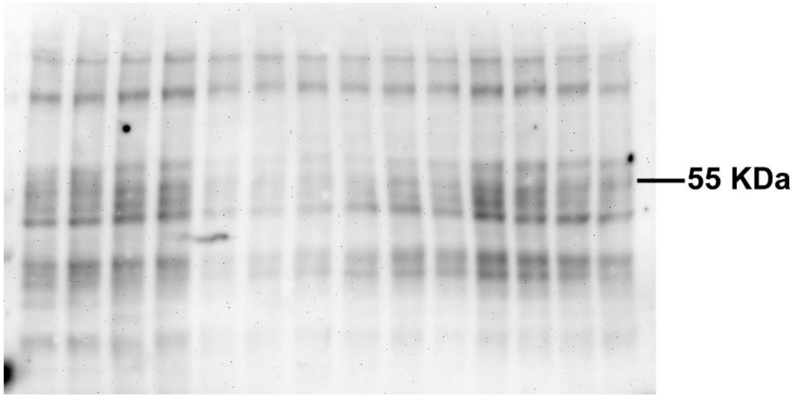

- **Na/K ATPase**

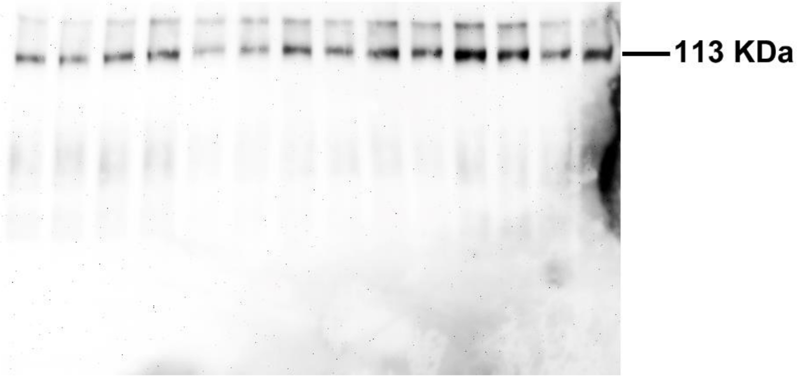

- **CD147 (cytosol)**

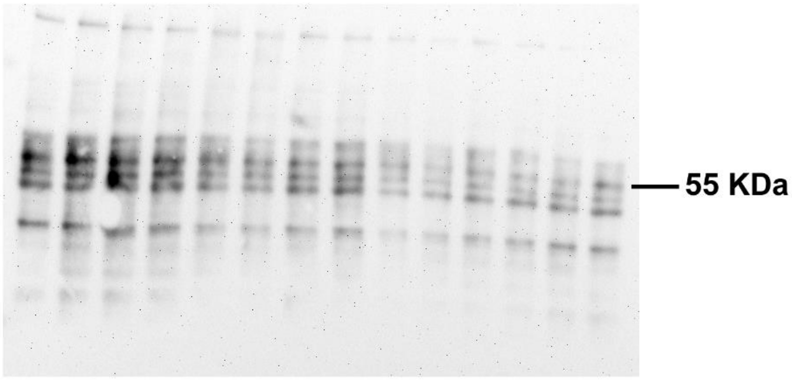

- **Actin**

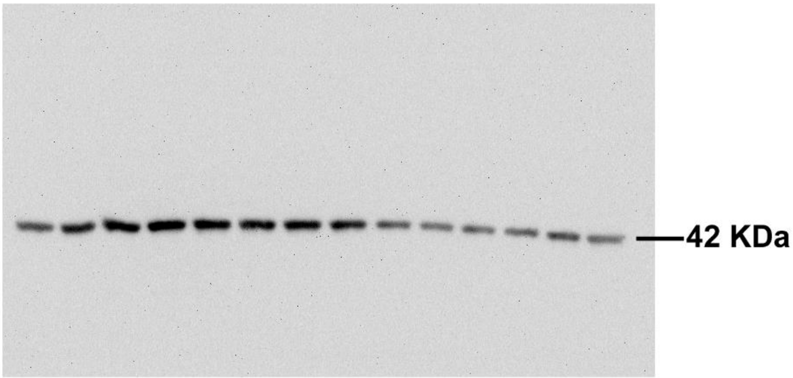

-Figure 3B

- **CD147 (membrane)**

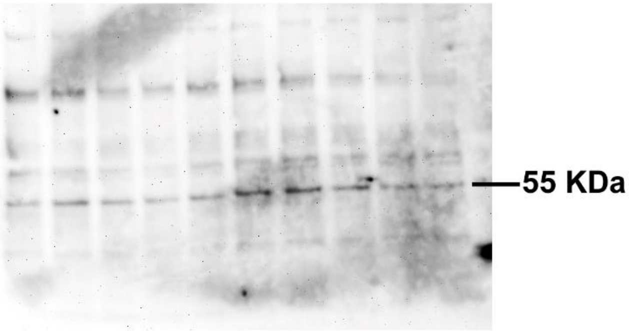

- **Na/K ATPase**

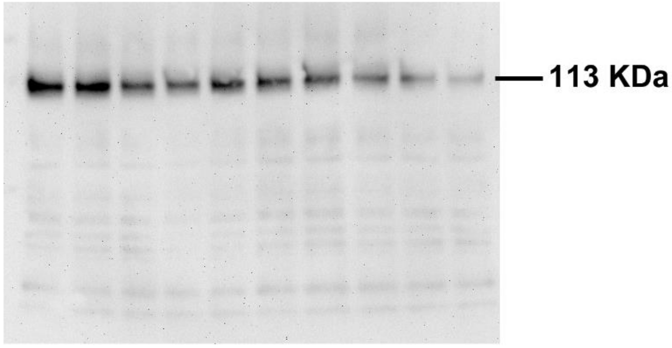

- **CD147 (cytosol)**

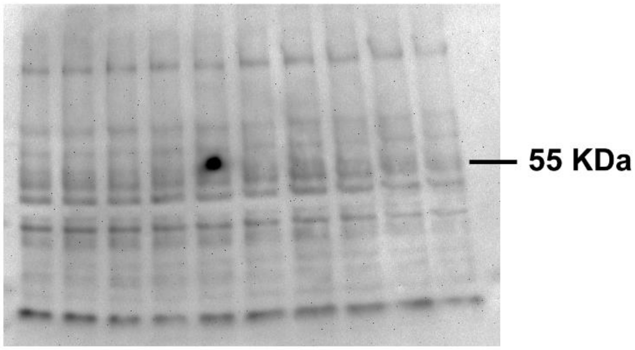

- **Actin**

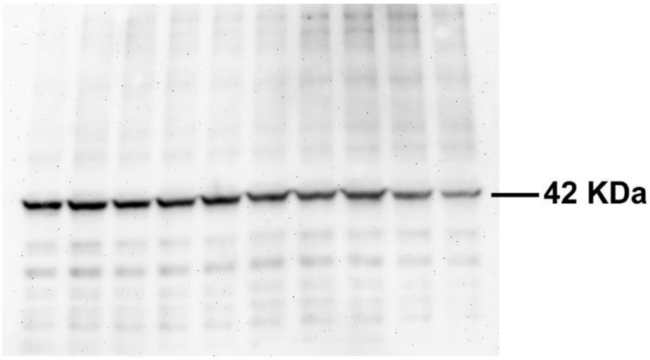

Figure 4A

- **pENOS (S1177)**

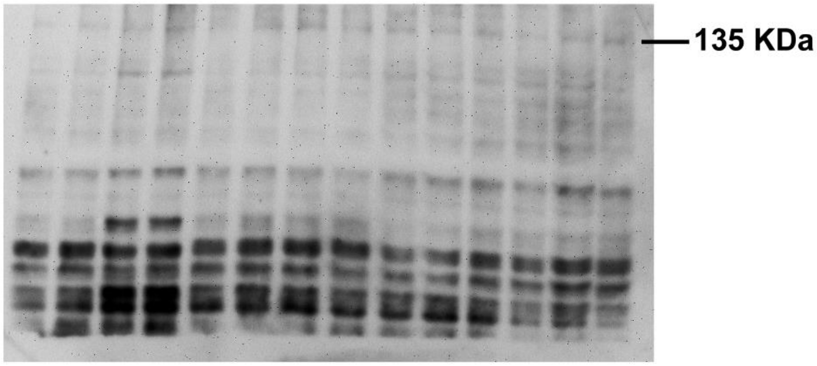

- **tENOS**

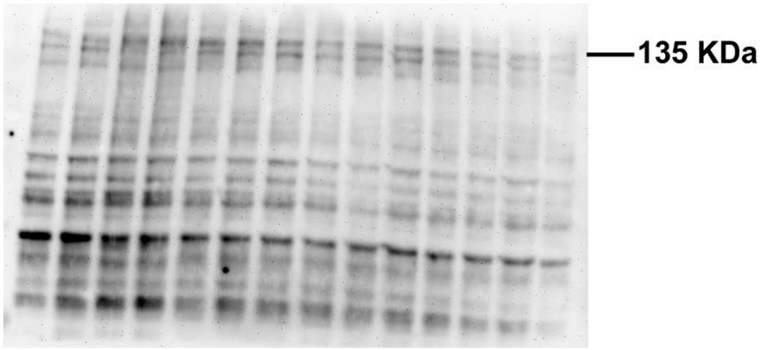

- **Actin**

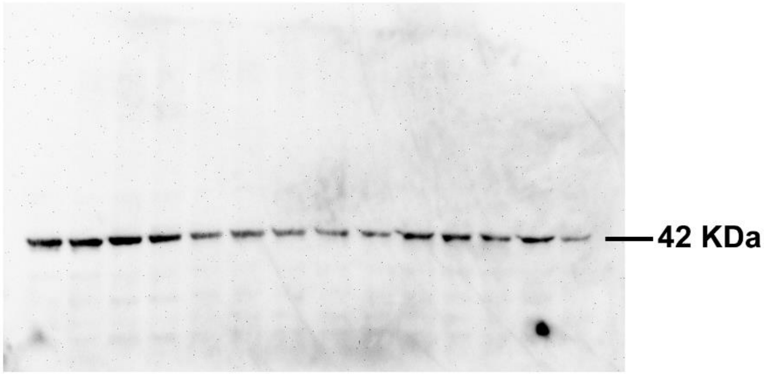

Figure 4B

- **pENOS (S1177)**

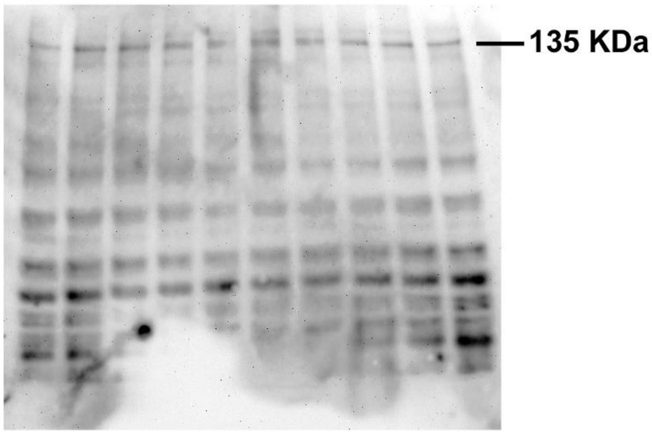

- **tENOS**

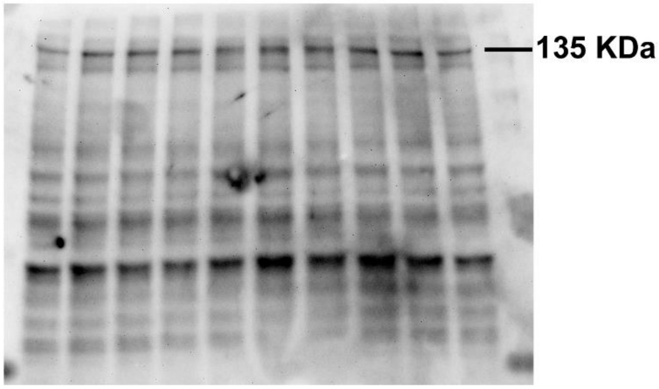

- **Actin**

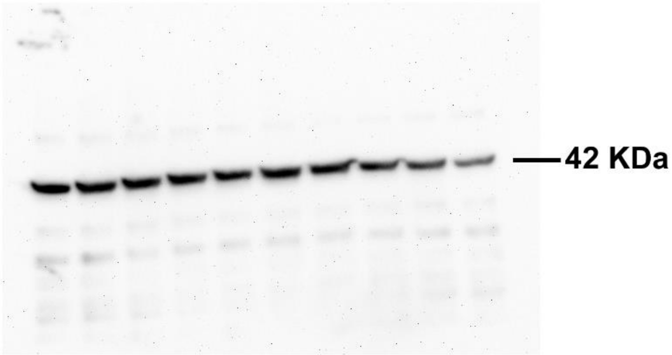

Figure 4C

- **NFkB-p65 (nucleus)**

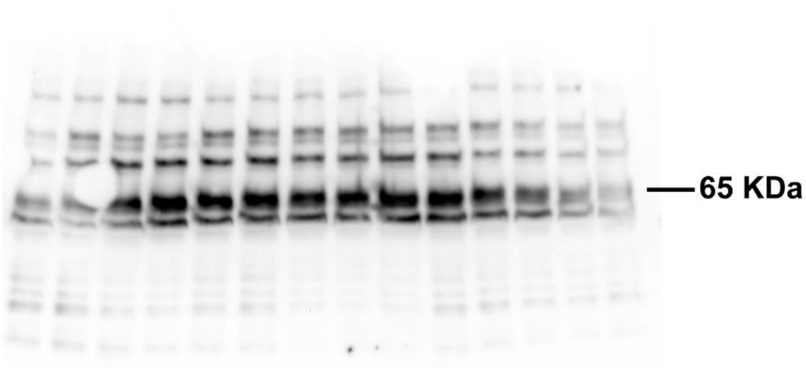

- **Lamin B1**

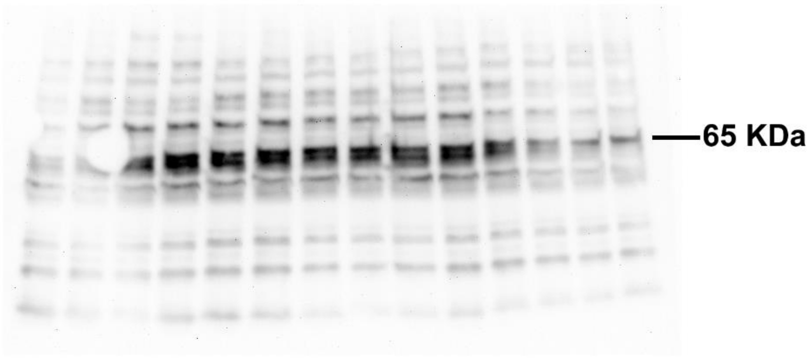

- **NFkB-p65 (cytosol)**

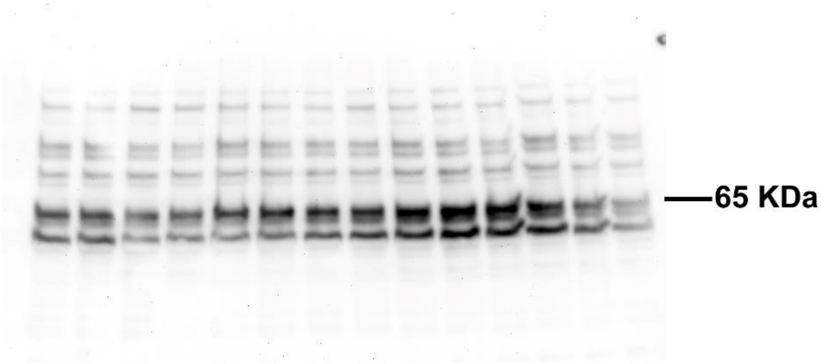

- **Actin**

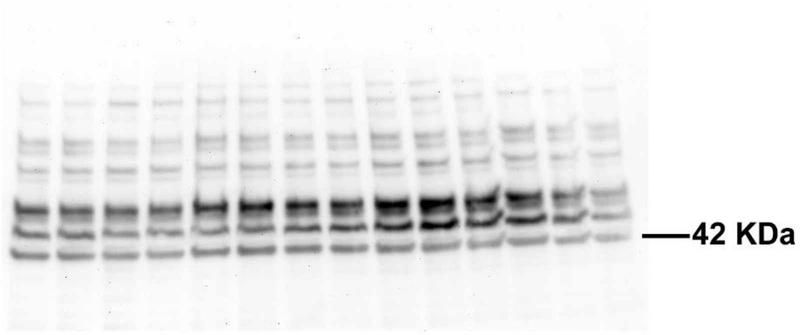

Figure 4D

- **NFkB-p65 (nucleus)**

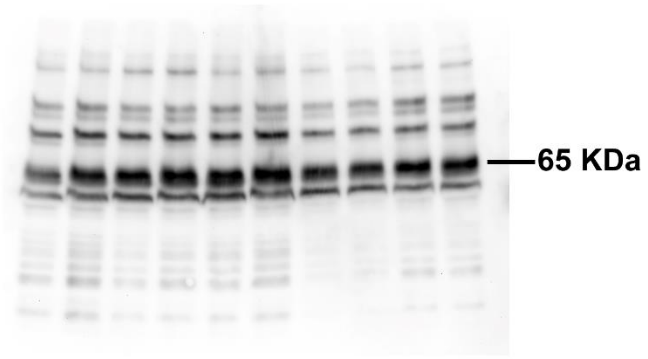

- **Lamin B1**

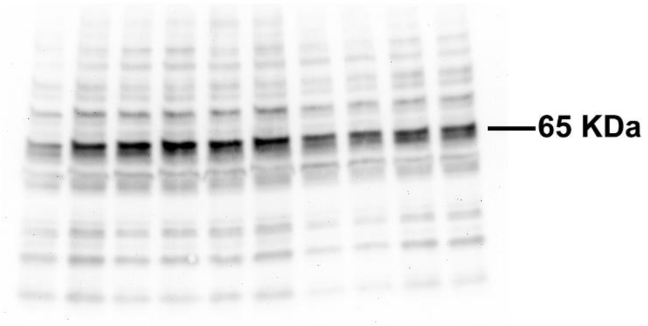

- **NFkB-p65 (cytosol)**

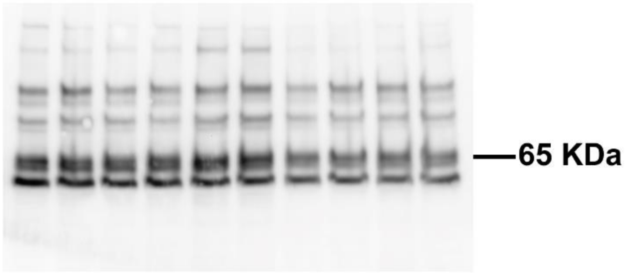

- **Actin**

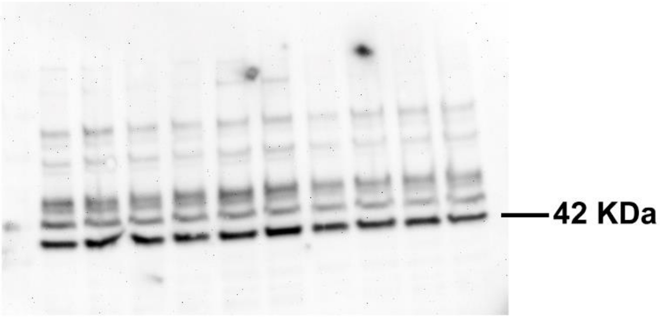

Figure 5A

- **iCypA**

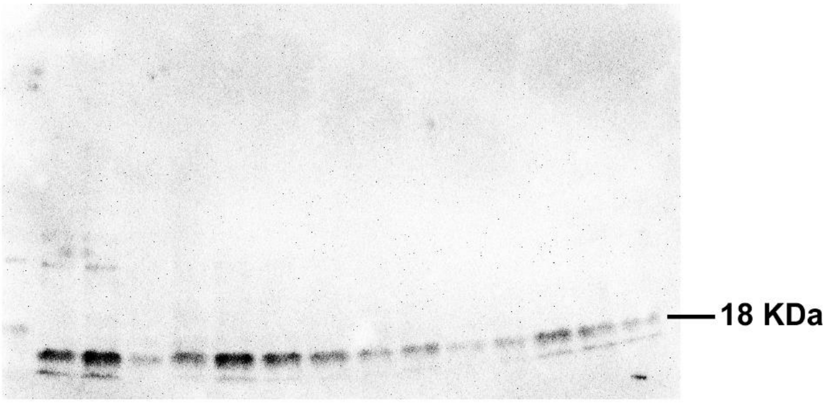

- **Actin**

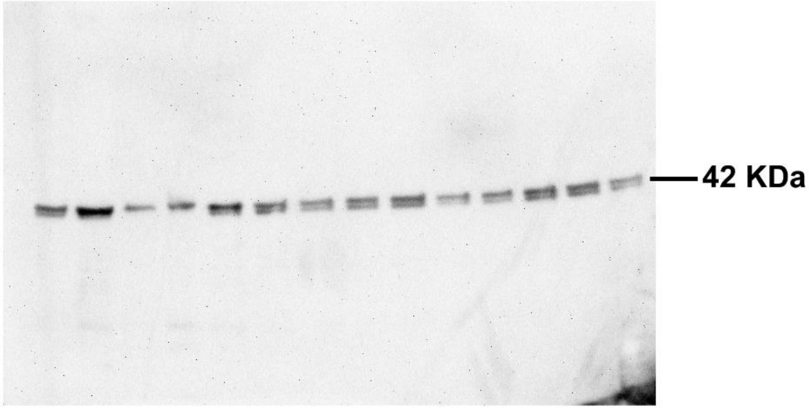

Figure 5B

- **iCypA**

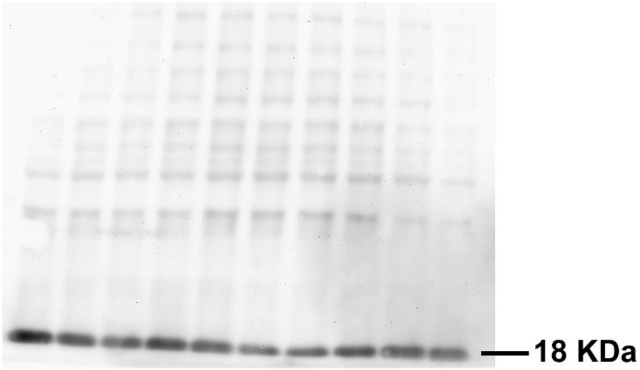

- **Actin**

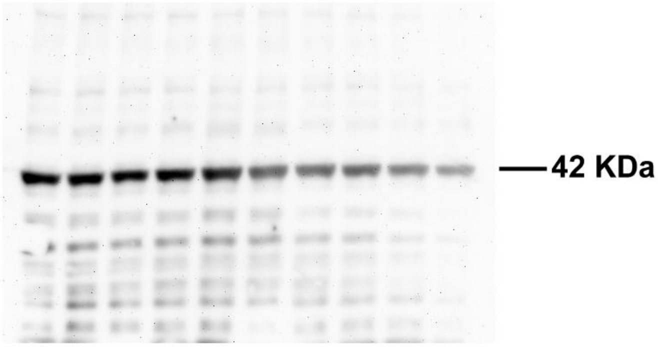

Figure 5C

- **iCypB**

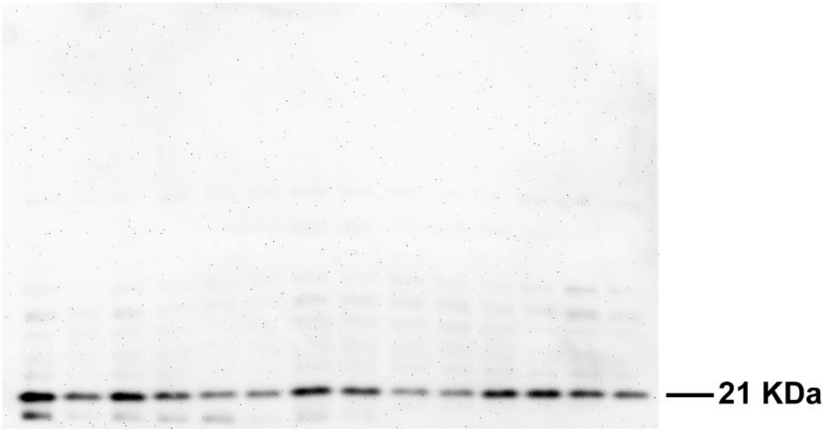

• **Actin**

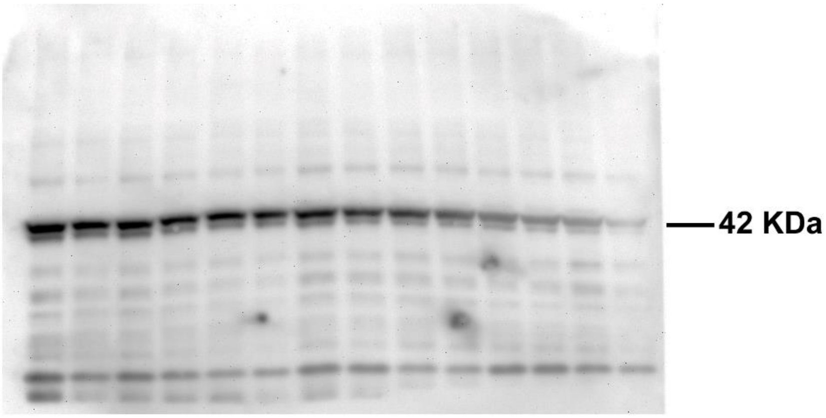

Figure 5D

• **iCypB**

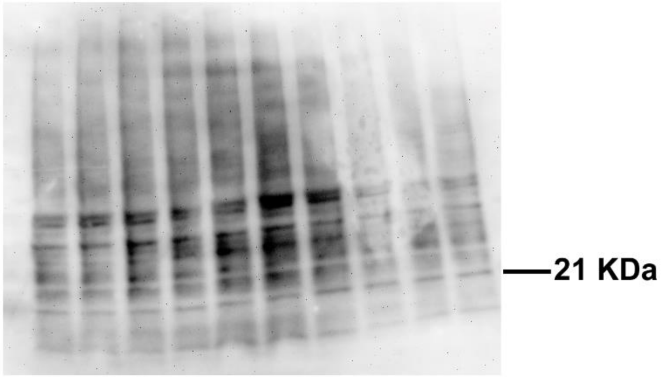

• **Actin**

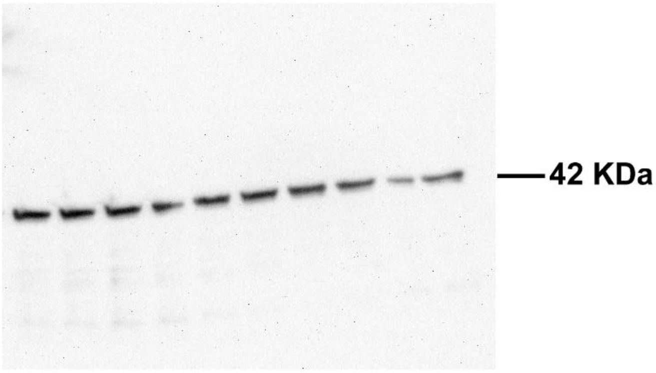

Figure 5E

- **iCypC**

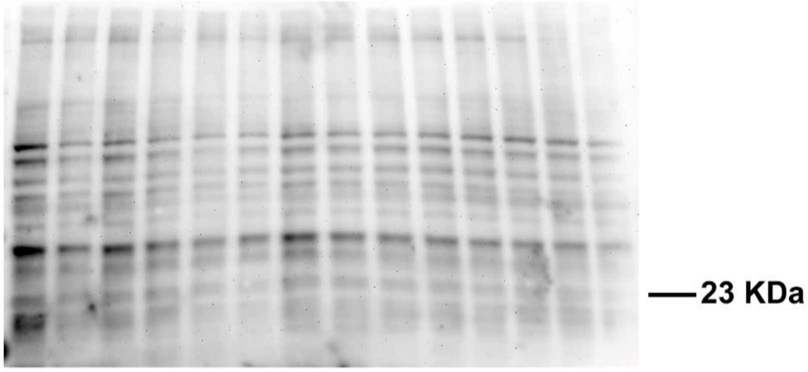

- **Actin**

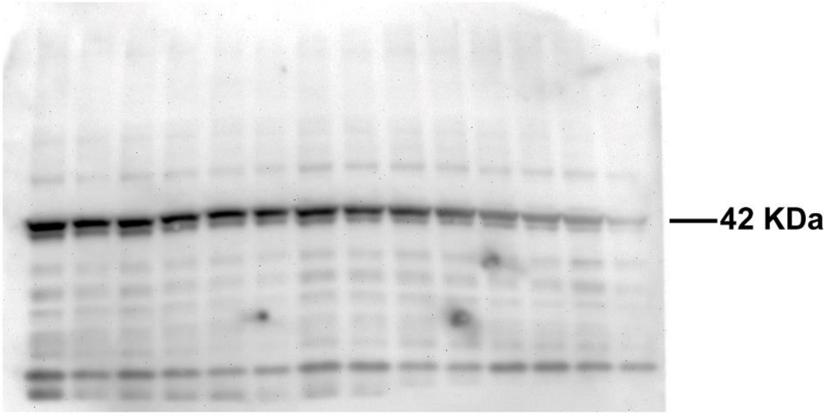

Figure 5F

- **iCypC**

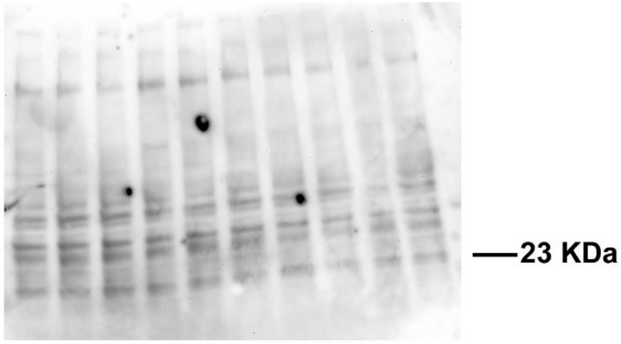

- **Actin**

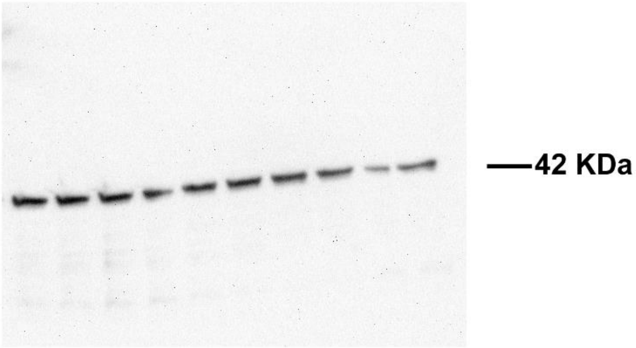

Figure 5G

- **iCypD**

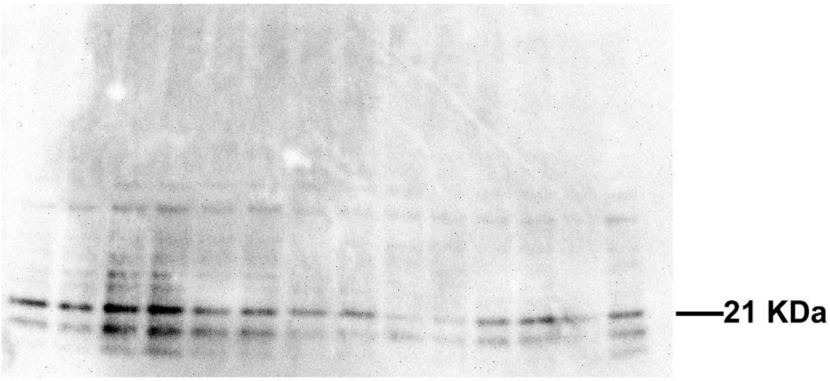

- **Actin**

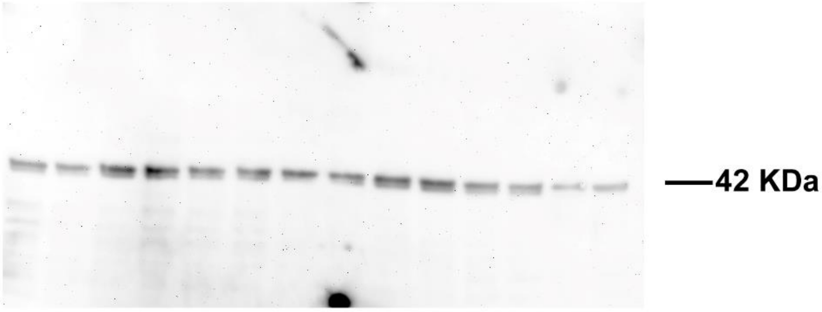

Figure 5H

- **iCypD**

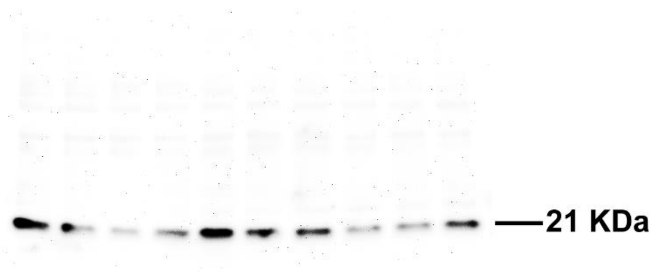

- **Actin**

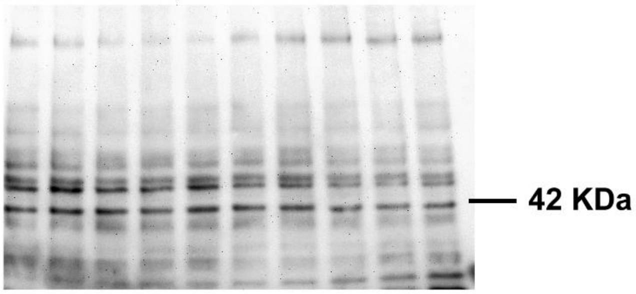

Supplement: Supplementary file 1 [file DataSheet1.pdf]
